# Editorial: The role of brain oscillatory activity in human sensorimotor control and learning: bridging theory and practice

**DOI:** 10.3389/fnsys.2023.1211763

**Published:** 2023-06-20

**Authors:** Elisa Tatti, Alberto Cacciola

**Affiliations:** ^1^City University of New York, School of Medicine, New York, NY, United States; ^2^Brain Mapping Lab, Department of Biomedical, Dental Sciences and Morphological and Functional Images, University of Messina, Messina, Italy; ^3^Center for Complex Network Intelligence (CCNI), Tsinghua Laboratory of Brain and Intelligence (THBI), Tsinghua University, Beijing, China; ^4^Department of Biomedical Engineering, Tsinghua University, Beijing, China

**Keywords:** sensorimotor system, motor control, brain oscillations, EEG, motor learning, beta ERD/ERS, gamma ERS, mu ERD

Neural oscillations, rhythmic patterns of oscillatory activity that occur both spontaneously and when the brain is engaged in a task, play a crucial role in the orchestration of neural communication within and across functional networks. Within the sensorimotor network, oscillations in the mu (8–13 Hz), beta (13.5–25 Hz), and gamma (30-90 Hz) frequency ranges are typically time-locked to movement onset and are characterized by a gradual amplitude decrease (Event-Related Desynchronization, ERD) or increase (Event-Related Synchronization ERS) during motor planning and execution. Although their functional role is still debated, mu, beta, and gamma oscillations are altered in several neuropsychiatric conditions (Peter et al., 2022), and are thought to be linked to sensorimotor control, learning, and plasticity (Pfurtscheller and Lopes da Silva, 1999; Engel and Fries, 2010; Ghilardi et al., 2021).

This Research Topic showcases investigations on the role of cortical oscillations in motor control and learning and the translational applicability of such knowledge. It comprises five articles spanning experimental and methodological investigations and a literature review.

Cho et al. investigated the effect of using a robotic orthosis coupled with functional electrical stimulation (FES) on mu and beta ERD in healthy young individuals. In the orthosis condition, participants performed finger extensions using a custom-made orthosis, whereas, in the combined conditions, extension movements occurred using both the orthosis and FES. From a kinematic perspective, the movement consisted of a dynamic extension phase and a static hold of the extension. Results showed stronger mu and beta ERDs during the dynamic compared to the static phase. Combined FES and orthosis led to a faster mu but not beta ERD, with no peak amplitude changes. No differences were observed in terms of beta rebound (Cho et al.). Overall, this study indicates that mu ERD is more sensitive than beta to muscle kinetics induced by FES, a finding that could have implications for neuromodulation and neurorehabilitation interventions.

While most of the research in motor control focuses on limb movements, Nakamura et al. explored postural instability, a disabling symptom in several neurological disorders, including Parkinson's disease (PD) and stroke. Cortical theta and beta ERD-ERS were examined during a perturbed upright stance to investigate supraspinal contributions to postural stabilization. Results show that, during a brief support-surface perturbation task, EEG activity displayed the typical beta ERD-ERS dynamics, and decreased theta over the primary motor area. Differently from what was observed for upper extremity movements, beta ERS was initiated before the postural recovery was completed and was sustained for a long duration, with minimal electromyographic activity. The authors interpreted beta ERS as reflecting either up-regulation of sensory inputs to sustain postural maintenance or the residual error in postural recovery. Thus, beta ERS might signal the active monitoring of the postural state until stability is reached, thus representing feedback-based sensory processing and/or attentional control. Further, theta ERD occurred in parallel with the beta rebound (Nakamura et al.), possibly suggesting a coordinated role with beta in the processing of sensory information.

Besides their role in the active control of movements, research is also unveiling the role of brain oscillations in learning. Acquiring or improving a sensorimotor skill depends on a series of processes leading to the creation and refinement of motor synergies, through plasticity mechanisms. In addition to the amount of practice and feedback type, other key factors influence the quality and amount of learning, including the structure of the implemented training. Mariman et al. explored the effects of two learning protocols: whole learning, which involves training on all skills included in a task simultaneously, and part learning, which segments the learning process into smaller, separately learned sub-components. Eighteen participants completed a video game that involves sensorimotor adaptations and skill learning using either whole or part learning. Results show that whole learning led to greater integration of different skill components and resulted in higher task performance. Furthermore, during epochs characterized by distorted kinematics, both groups exhibited less evoked activity in central and posterior areas. In contrast, during the epochs characterized by distorted dynamics, only the whole learning group demonstrated a decrease in ERP amplitude in the anterior and central-posterior electrodes. Altogether, the authors concluded that neural correlates of kinematic and dynamic control were modulated by sensorimotor learning, and the practice type affected the quality of learning (Mariman et al.).

Understanding the neural mechanisms behind motor learning can have important translational applications to improve motor and sports performance, enhance rehabilitation protocols, and support the development of new technologies to support the independence and functioning of individuals with sensorimotor injuries.

In this regard, Leonardi et al. provide an overview of the existing randomized controlled trials exploring brain rhythms as predictors of stroke recovery. Thirteen studies involving 346 patients were included in the review and converged in showing acute alterations in the alpha, beta, and gamma frequency ranges. Specifically, the authors report increased alpha and beta oscillatory activity and disruption of gamma oscillations. Further, they found a correlation between post-stroke oscillatory activity and functional recovery in the chronic phase, with greater alpha ERD being associated with improved functional outcomes. Also, greater beta and gamma power in the ipsilesional hemisphere, but not contralesional beta, was positively associated with recovery (Leonardi et al.).

In addition, cortical oscillations may be helpful in supporting the outcome of deep brain stimulation (DBS) treatment. Sand et al. assessed the use of electroencephalography (EEG) as a tool for subthalamic nucleus (STN) DBS lead localization in 17 PD patients. Machine learning models were used to identify the location of the stimulating leads based on the extracted cortical stimulation-evoked potential features. STN-evoked responses over the medial and ipsilateral frontocentral areas reliably predicted the location of STN DBS electrodes placed in the Zona incerta and the dorsolateral, and ventromedial regions of the STN. As the localization of electrode contacts is crucial for the efficacy of the DBS treatment, using cortical evoked responses to estimate the position of DBS electrodes offers a promising non-invasive, and cost-effective approach for monitoring DBS procedures.

In conclusion, the studies included in this Research Topic provided valuable insights into the role of brain oscillations in human sensorimotor control and learning, as well as the translational applications of such knowledge to enhance motor performance and address disorders characterized by sensorimotor dysfunction. By studying brain oscillations, research can foster our understanding of the neural correlates of sensorimotor control and provide insights into the mechanisms behind diseases characterized by sensorimotor dysfunction, potentially aiding the diagnosis, predicting the functional outcome, and developing rehabilitative interventions and brain-computer interface devices to restore or compensate for the impaired sensorimotor functions.

## Author contributions

ET and AC wrote and reviewed the submitted editorial. All authors contributed to the article and approved the submitted version.

## References

[B1] Engel A. K. Fries P. (2010). Beta-band oscillations-signalling the status quo? Curr. Opin. Neurobiol. 20, 156–165. 10.1016/j.conb.2010.02.015 20359884

[B2] Ghilardi M. F. Tatti E. Quartarone A. (2021). Beta power and movement-related beta modulation as hallmarks of energy for plasticity induction: Implications for Parkinson's disease. Park. Relat. Disord. 88, 136–139. 10.1016/j.parkreldis.2021.05.018 34144879

[B3] Peter J. Ferraioli F. Mathew D. George S. Chan C. Alalade T.. (2022). Movement-related beta ERD and ERS abnormalities in neuropsychiatric disorders. Front. Neurosci. 16, 1–19. 10.3389/fnins.2022.1045715 36507340 PMC9726921

[B4] Pfurtscheller G. Lopes da Silva F. H. (1999). Event-related EEG/MEG synchronization and desynchronization: basic principles. Clin. Neurophysiol. 110, 1842–1857. 10.1016/S1388-2457(99)00141-8 10576479

